# Hyperplastic thymus with increased angiogenesis is correlated with elevated serum thyroglobulin level in differentiated thyroid cancer patients with TENIS syndrome

**DOI:** 10.18632/oncotarget.23281

**Published:** 2017-12-15

**Authors:** Guangjian Zhang, Rui Gao, Yuanbo Wang, Yan Liu, Juan Li, Xi Jia, Yiqian Liang, Aimin Yang

**Affiliations:** ^1^ Department of Thoracic Surgery, The First Affiliated Hospital of Xi’an Jiaotong University, Xi’an 710061, China; ^2^ Department of Nuclear Medicine, The First Affiliated Hospital of Xi’an Jiaotong University, Xi’an 710061, China

**Keywords:** differentiated thyroid cancer, TENIS syndrome, integrin α_v_β_3_, thymus, RGD peptide

## Abstract

**Aims:**

To investigate the association between angiogenetic activity of hyperplastic thymus and serum thyroglobulin (Tg) level in differentiated thyroid carcinoma patients with thyroglobulin (Tg)-elevated Negative Iodine Scintigraphy (TENIS) Syndrome.

**Methods:**

A cohort of 30 consecutive patients who underwent total thyroidectomy followed by radioiodine ablation and had TENIS syndrome received integrin α_v_β_3_ targeted imaging with ^99m^Tc-HYNIC-PEG4-E[PEG4-c(RGDfk)]2 (^99m^Tc-3PRGD2). The correlation of angiogenetic activity of the thymus and the serum Tg levels was evaluated in patients with enlarged thymus.

**Results:**

Enlarged thymus was detected in 9 out of the 30 TENIS patients and all hyperplastic thymus showed an increased accumulation of the tracer (median tumor/background ratio: 2.8). Five of them had only mediastinal uptake and surgical removal of the mediastinal mass in one provided histopathologic evidence of thymic tissue. The other four were not assigned further treatment and were free of disease in the follow-up, though their stimulated Tg levels consistently increased. Four out of the 9 patients showed ^99m^Tc-3PRGD2 uptake outside the mediastinum were assigned surgery followed by radioiodine treatment. Their stimulated Tg levels decreased after iodine ablation, but not drop back to normal. A significant linear correlation was observed between serum Tg levels and the degree of angiogenesis in the hyperplastic thymus.

**Conclusions:**

The angiogenetic activity in hyperplastic thymus was related with the consistently elevated serum Tg levels in TENIS syndrome patients. Based on the existing literature and current data, we propose further intervention for patients with RGD uptake outside thymus, while close follow-up for patients with only mediastinal uptake.

## INTRODUCTION

Differentiated thyroid cancer (DTC) accounts for 1% of all malignant tumors, and has shown a rapid rise in the past several years [1]. Total thyroidectomy followed by iodine-131 (^131^I) ablation is recommended for the initial treatment of DTC patients, with serial serum thyroglobulin (Tg) tests and imaging measurements as follow-up [2]. A situation as there is no radiologically or clinically evident disease, but Tg levels remain detectable or even significantly elevated (TENIS syndrome) is common in clinical practice [3]. The probable explanation for such condition might be that lesion is too small to image but large enough to secret Tg [4]. Therefore, an empirical ^131^I ablation or surgery was suggested for TENIS syndrome patients with Tg level of more than 10 μg/L [5]. However, as suggested by recent reports, there is a possibility that the detected Tg was produced by other benign sources [6, 7]. Apparently, the recognition of physiological Tg secretion is critical for patients with the TENIS syndrome to avoid unnecessary treatments.

The ability to concentrate iodide represents a critical step in the production of Tg [8]. Iodide uptake has been observed in various extra thyroidal tissues in post-surgery DTC patients, including thymus, salivary gland, gastric mucosa, and mammary gland [9]. Some recent studies have identified thymic radioiodine uptake as a frequent cause of a false-positive mediastinal focus on the whole-body ^131^I scan (^131^I WBS) of post-surgery DTC cases [10, 11]. As elevated Tg level is frequently observed in DTC patients with thymic hyperplasia, thymus has been suggested as a potential source for physiological Tg secretion [12, 13]. It is noteworthy that studies revealed a disappearance of the Tg elevation after thymus resection in DTC patients who had previously undergone total thyroidectomy and ^131^I therapy [14]. The “thyroid follicle-like” Hassall bodies in the hyperplastic thymus, which could localize iodine, might be responsible for iodine uptake and Tg secretion in thymus [15].

Thymic endocrine activity was reported to be strongly influenced by neuroendocrine signals, especially thyroid hormones. Thyroid hormones exert their action on the epithelial cells of the thymus deputed to synthesize and secrete thymic peptides [16]. As a result, functional evaluation of the enlarged thymus is essential for understanding its roles in the TENIS syndrome [17]. To our knowledge, no study has investigated the functional changes of hyperplastic thymus in patients with TENIS syndrome. Hence, in this study, we investigated the angiogenetic degree of hyperplastic thymus and analyzed its association with serum Tg level in patients with TENIS syndrome. Technetium 99m (^99m^Tc)-dimeric cyclic arginine-glycine-aspartic acid (RGD) peptides with three polyethylene glycol spacers (^99m^Tc-3PRGD2) single photon emission computed tomography (SPECT)/X-ray computed tomography (CT) scanning, which has long been adopted in evaluating the angiogenesis in studies through targeting integrin α_v_β_3_, was used in the present study to measure the angiogenetic activity of hyperplastic thymus.

## RESULTS

All 30 patients with TENIS syndrome had initially received total thyroidectomy and high-dose radioiodine ablation. The median cumulative radioiodine therapy dose was 8.14 GBq (range, 3.7–24.05 GBq). The final diagnostic radioiodine scan was negative along with elevated Tg levels (median stimulated Tg, 108.88 ng/mL; range, 13.5–480 ng/mL) and a negative neck US and chest radiography. The demographic and related clinical data of all TENIS syndrome patients are listed in Table 1.

**Table 1:** Characteristics of the patients with TENIS syndrome (*n* = 30)

Characteristic	Median (range)
Age (years)	42 (18–61)
Gender (*n*)	
Female	26
Male	4
Histology (*n*)	
Papillary thyroid carcinoma	26
Follicular thyroid carcinoma	4
Thyroglobulin (ng/mL)	108.88 (13.5–480)
TSH (uIU/mL)	76.78 (47.1–100)
Tg Ab (ng/mL)	3.26 (2.53–6.63)
Diameter (cm)^*^	0.72 (0.1–3.0)
Lymph nodes metastasis^†^	8.78 (0–34)

TSH: thyroid-stimulating hormone; Tg Ab: serum thyroglobulin antibody; ^*^the diameter of the primary thyroid tumor; ^†^number of lymph nodes dissected in surgery shown by pathology studies as metastatic lesions.

**Figure 1:**
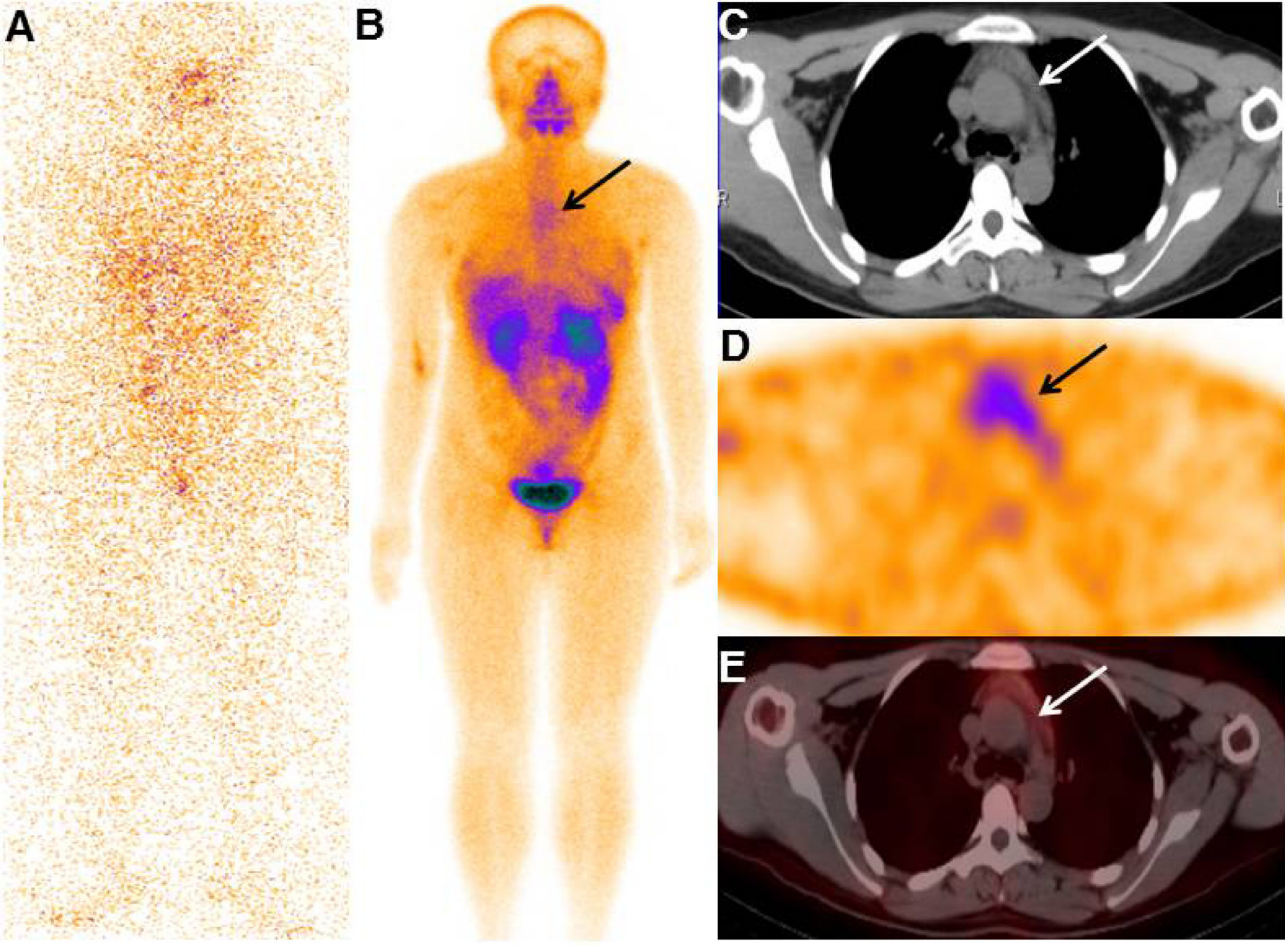
Typical image of the TENIS patient with enlarged thymus that accumulating ^99m^Tc-3PRGD2 The final diagnostic radioiodine scan was negative (**A**), while whole-body projection SPECT image showed increased ^99m^Tc-3PRGD2 uptake in the mediastinal region (**B**, arrow). SPECT/CT images showed increased radiopharmaceutical uptake corresponding to the enlarged thymus in the patient (**C**–**E**, arrows; from the first to the third row).

In 9 of 30 patients (30.0%), co-registered diagnostic CT scan for ^99m^Tc-3PRGD2 SPECT/CT revealed an enlarged thymus, a finding that was compatible with the ^99m^Tc-3PRGD2 uptake observed on SPECT/CT images, i.e. all enlarged thymus showed an increased accumulation of the tracer (Figure 1). This group was comprised of seven female and two male papillary thyroid carcinoma patients with a median age of 29 years (range, 19–38 years) (Table 2). Five of the 9 patients with hyperplastic thymus (55.56%) showed only mediastinal uptake of ^99m^Tc-3PRGD2 in SPECT/CT, and the remaining 4 patients (44.44%) also had uptakes outside the mediastinum. These 9 patients were followed up for 11–24 months (median 16 months) after the ^99m^Tc-3PRGD2 SPECT/CT scan was performed.

**Table 2:** Characteristics of the patients with mediastinum uptake of ^99m^Tc-3PRGD2 (*n* = 9)

No.	Gender	Age (Yrs)	Lymph nodes^*^	^131^I dose (GBq)^†^	Tg before (ng/mL)^‡^	Tg last (ng/mL)^§^	RGD uptake	Follow-up (months)	Disease status (5)
1	F	24	6	11.1	62.2	18.7 / NT	L	20	DF
2	F	32	16	9.25	33.3	28.3 / NT	L	16	DF
3	F	28	4	4.81	58.5	43.3 / NT	L	17	DF
4	M	29	7	8.14	19.5	10.5/1.75	L	12	DF
5	M	29	19	11.1	26.1	11.2/NT	L	11	DF
6	F	19	13	19.98	15.5	18.1 / 4.72	M	18	MD
7	F	23	5	9.25	20.3	18.3 / NT	M	24	DF
8	F	43	3	14.8	25.1	19.2 / NT	M	13	DF
9	F	38	31	10.36	18.2	14.6 / NT	M	14	DF

F: Female; M: Male; Yrs: years; Tg: serum thyroglobulin; mCi: millicuries; L: limited to mediastinal thymus uptake; M: metastatic to outside uptake; DF: disease free; MD; metastatic disease; Follow-up: duration of follow-up since the performance of ^99m^Tc-3PRGD2 SPECT/CT; NT: not detectable; ^*^lymph nodes dissected in surgery; ^†^cumulative dose since initiative ^131^I ablation; ^‡^serum TSH stimulated Tg levels before SPECT/CT scan; ^§^serum stimulated/suppressed Tg levels at last follow-up.

**Figure 2:**
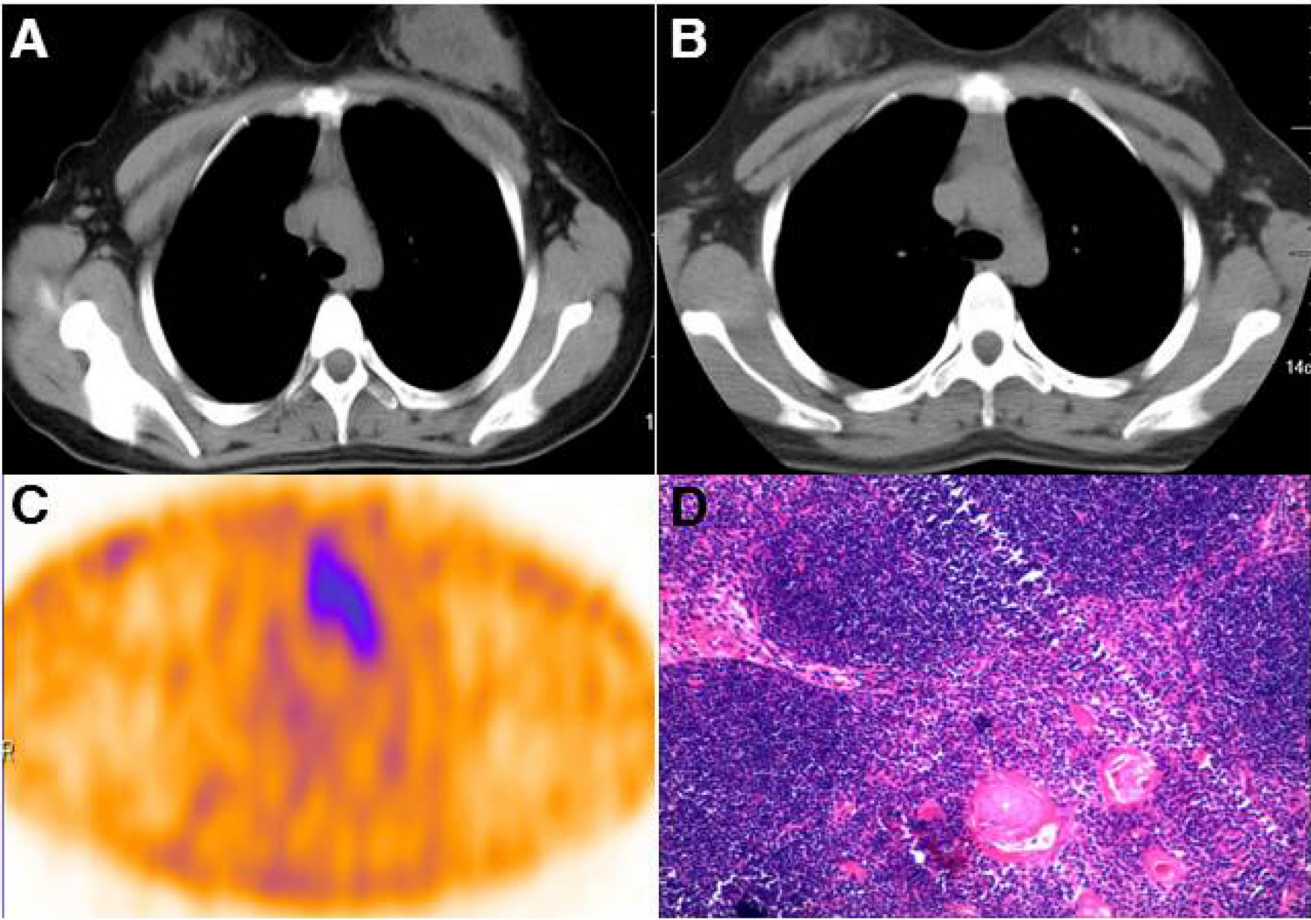
Surgical removal of the mediastinal mass provided histopathologic evidence of thymic tissue Chest computed tomography showed a large anterior mediastinal mass behind the sternum in Patient 1 (**A**). The volume of the mass increased gradually (**B**) and showed increased 3PRGD2 uptake on SPECT/CT scan (**C**). The pathological examination revealed hyperplastic thymic tissue in the mass (**D**).

The 5 patients who had only mediastinal uptake had serum stimulated Tg levels ranging from 33.3 to 62.2 ng/mL before ^99m^Tc-3PRGD2 SPECT/CT scan (Patients 1–5, Table 2). One patient (Patient 1) had serial CT scans showed progressive thymus enlargement (Figure 2); therefore, this patient underwent mediastinal dissection. Histologic examination of resected specimen revealed hyperplastic thymic tissue (Figure 2). The serum Tg level of Patient 1 was significantly decreased after surgery. No any other treatment was carried out between the surgery and the laboratory test, and she was free of disease during a 20-month follow-up (Table 2). In other 4 patients (Patient 2 and 5) with only mediastinal uptake, CT scanning revealed moderately enlarged thymus. Treatment of these 4 patients consisted of inhibition of TSH secretion by levothyroxine (target TSH level, <0.07 uIU/mL) without performing additional surgery or radioiodine treatment. There were no clinical signs of recurrence or metastasis observed during their follow-up; however, their serum stimulated Tg levels were still remarkably elevated by the last follow-up (Table 2).

**Figure 3:**
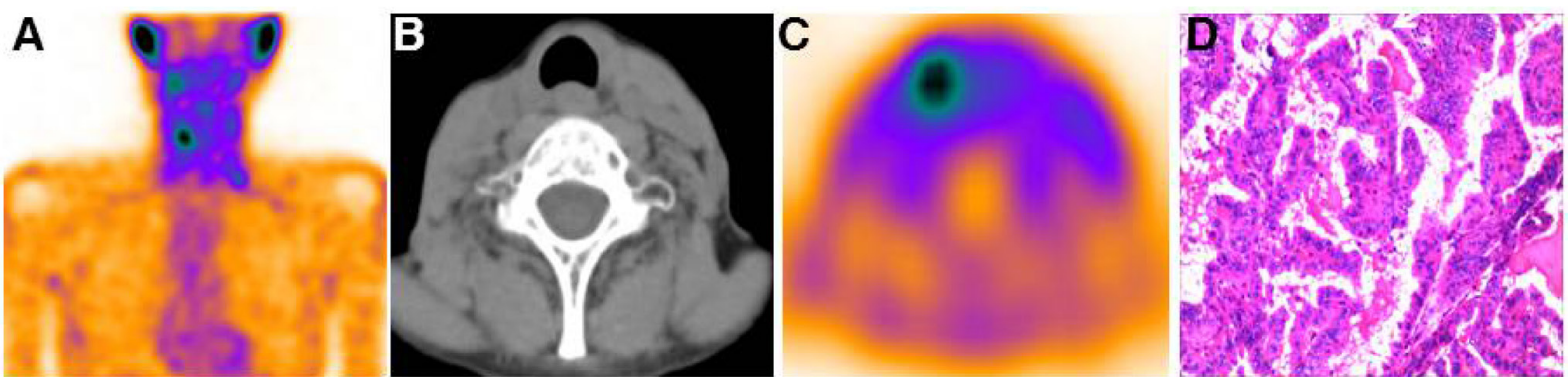
^99m^Tc-3PRGD2 uptake outside the thymus was documented cervical lymph nodes metastasis The ^99m^Tc-3PRGD2 planetary images of Patient 7 showed increased uptake of the tracer outside mediastinal region (**A**). The SPECT/CT images demonstrated increased radiopharmaceutical uptake corresponding to the cervical lymph nodes (**B** and **C**). The patient underwent cervical lymph node dissection and histopathology results documented metastasis disease from DTC (**C**).

Four patients who had mediastinal uptake of 3PRGD2 and uptake outside the mediastinum are also presented in Table 2 (Patients 6–9). Their TSH-stimulated Tg levels ranged from 15.5 to 25.1 ng/mL before ^99m^Tc-3PRGD2 SPECT/CT scanning. Treatment of these patients was based on the existence of extra-mediastinal uptake of ^99m^Tc-3PRGD2. In Patient 6–8, whose contrast enhanced CT results confirmed the presence of cervical and mediastinal lymph node metastasis, surgery followed by empirical ^131^I treatment (5.55 GBq, 5.55 GBq and 6.29 GBq, respectively) was performed (Figure 3). Their Tg levels were decreased after radioiodine treatment; however, the stimulated Tg levels were still elevated, and even higher at last follow-up in Patient 6 (Table 2). By the end of the study, metastatic lymph node disease was indicated by cervical US in Patient 6, while no signs of recurrence were detected in Patient 7 and 8. The last patient (Patient 9) showed possible recurrent disease in the thyroid bed on ^99m^Tc-3PRGD2 SPECT/CT. A dose of 5.55 GBq ^131^I treatment was carried out in Patient 9, and similar to the other 3 patients, stimulated serum Tg level of this patient did not drop back to the expected level (≤2 ng/mL) after radioiodine treatment (Table 2).

**Figure 4:**
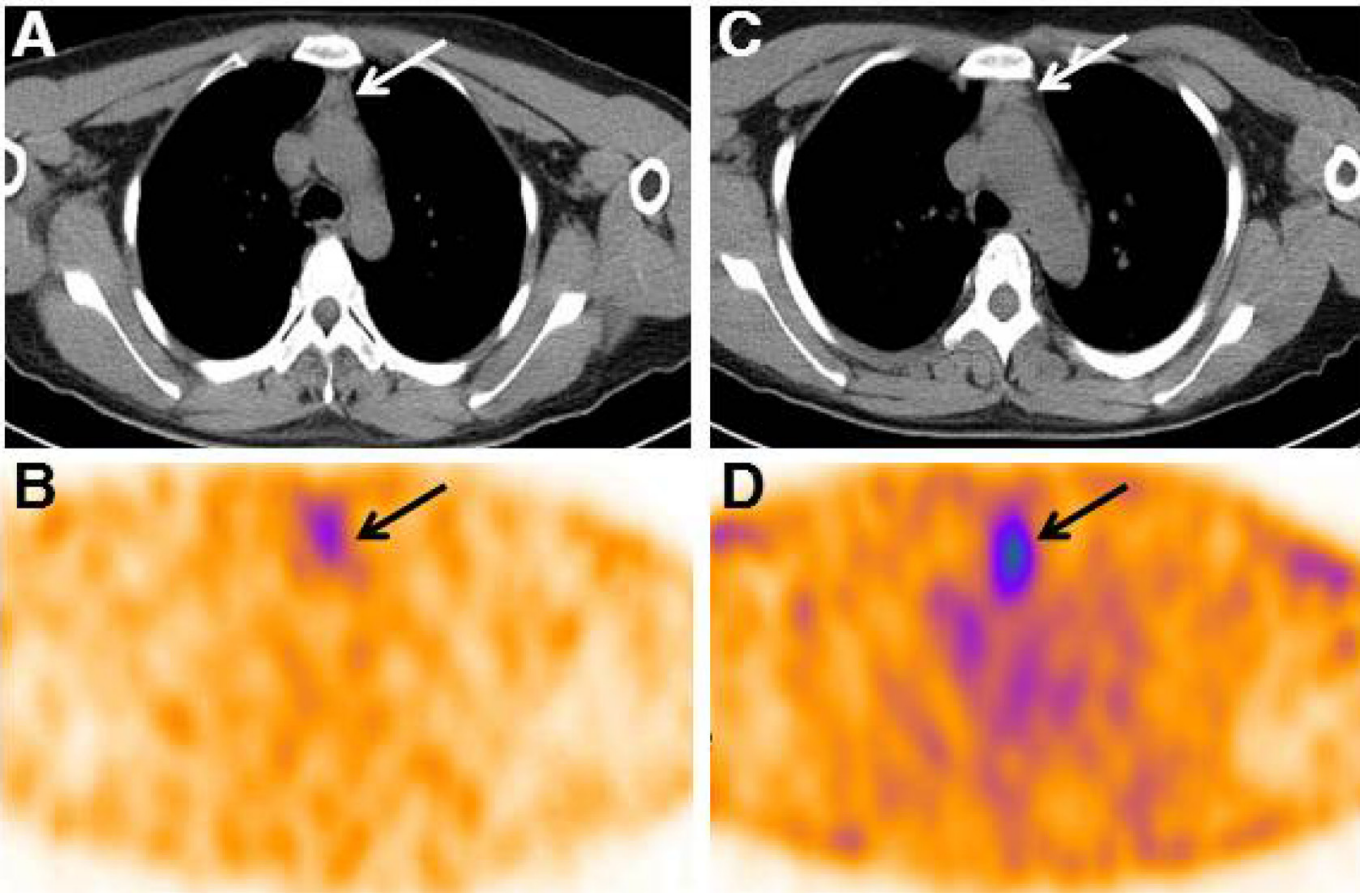
Two representative ^99m^Tc-3PRGD2 SPECT/CT images of TENIS patients with different serum Tg levels (**A**–**B**) Patient 9 with stimulated Tg of 18.2 ng/mL demonstrated a uptake ratio of 1.8 in thymus; (**C–D**), Patient 3 with stimulated Tg of 58.5 ng/mL showed a T/B ratio of 3.2 in thymus.

**Figure 5:**
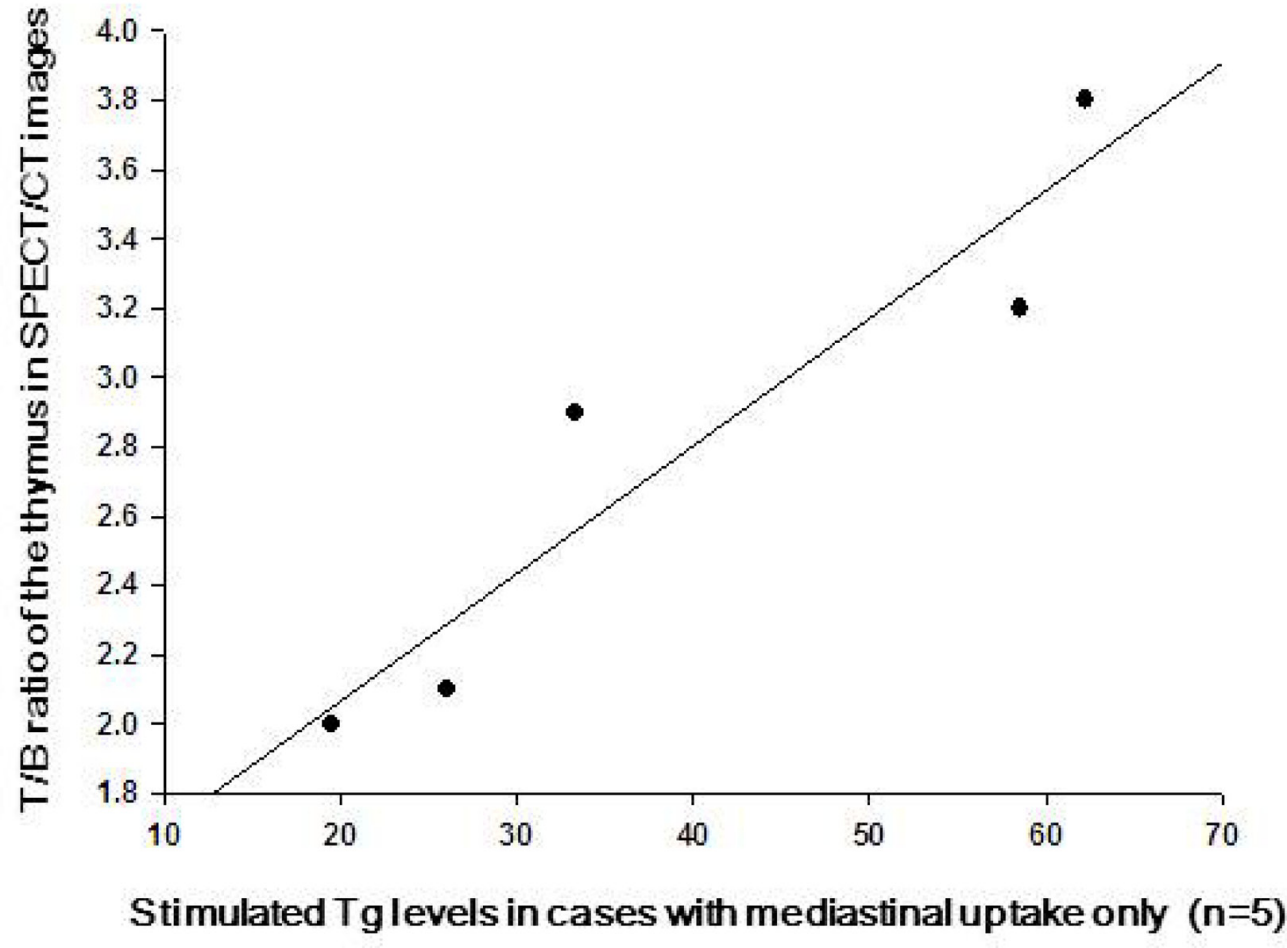
The correlation of the TSH-stimulated serum Tg levels and the angiogenesis activity of the hyperplastic thymus in cases with mediastinal uptake only Typical linear plots indicated that the TSH-stimulated serum Tg levels were significantly correlated with the Tumor/Background ratios of the enlarged thymus on ^99m^Tc-3PRGD2 SPECT/CT (*n* = 5).

The detailed description of the enlarged thymus was presented in Table 3. Most of the enlarged thymus presented as a shape of arrowhead with smooth margins. Because thymic thickness has been shown to be a more consistent indicator of thymic enlargement, we evaluated the relationship of thymic lobe thickness with serum stimulated Tg levels in these patients. However, no significant correlation was found between thymic lobe thickness and Tg levels. The median T/B ratio of thymus in SPECT/CT images was 2.8 (range 1.8–3.8) in the 9 TENIS syndrome patients with enlarged thymus (Figure 4). It is of note that a significant linear correlation was observed between serum Tg levels and the T/B ratios in patients with only thymus uptake (Pearson *r* = 0.939, *P* = 0.018, *n* = 5, Figure 5). To tentatively exclude the influence of the metastatic lesions on Tg levels, the post-treatment Tg levels of the 4 patients with metastatic disease, together with Tg levels of 5 patients with only mediastinal uptake, were used to analyze the correlation between serum Tg levels and the T/B ratios. A positive correlation was still found between the angiogenesis condition of the thymus and the Tg levels in 9 TENIS patients (Pearson *r* = 0.751, *P* = 0.020, *n* = 9).

**Table 3:** Characteristics of the enlarged thymus (*n* = 9)

No.	Volume dimensions (mm)			Shape	Margins	Internal characteristics
	thickness	transverse	craniocaudal			
1	25.3	30.1	25.1	quadrilateral	smooth	homogeneous
2	15.9	57.6	36.8	arrowhead	smooth	inhomogeneous
3	14.8	32.4	23.5	arrowhead	multi-lobular	inhomogeneous
4	15.4	51.8	40.0	arrowhead	smooth	homogeneous
5	14.0	31.2	17.7	arrowhead	smooth	homogeneous
6	15.0	18.2	15.9	arrowhead	smooth	inhomogeneous
7	15.4	59.4	35.3	arrowhead	smooth	homogeneous
8	13.2	23.9	16.9	rounded	multi-lobular	inhomogeneous
9	15.6	16.6	15.4	quadrilateral	smooth	inhomogeneous

## DISCUSSION

Elevated Tg levels coupled with a negative radioiodine scan present problems for the diagnosis and treatment of recurrent DTC [3]. Fluorine-18-fluorodeoxyglucose (^18^F-FDG) positron emission tomography/X-ray computed tomography (PET/CT) is an established imaging modality in cases with TENIS syndrome [18–20]. However, the low probability of obtaining a positive FDG PET result in TENIS patients with sTg levels lower than 30 ng/mL may hamper its clinical use [19, 20]. Previous studies have focused on the ability of ^99m^Tc-PEG4-E [PEG4-c (RGDfK)]2 (^99m^Tc-3PRGD2) SPECT/CT to specifically image metastatic lymph nodes in DTC patients [21, 22]. Our study, as far as we know, is the first one focused on its application in evaluating the physiological Tg secretion sources in the follow up of DTC patients. In the present study, 9 out of 30 DTC patients with TENIS syndrome presented upregulated angiogenesis in their enlarged thymus. Surgical removal of the mediastinal mass in 1 patient with progressive thymic hyperplasia provided histopathologic verification of thymic tissue, without any evidence of thyroidal or neoplastic cells. This patient had a significant decrease in the level of Tg after surgery, without any other treatment being performed between the surgery and the laboratory evaluation. This finding arises the possibility of thymic tissue as a potential source of benign production of Tg in TENIS syndrome patients. Thymus has been regarded as a benign source of Tg secretion for a long time. Recent studies have demonstrated the thymic Tg expression in embryonic mice and adult rats [23, 24]. Besides, Sospedra *et al.* found Tg transcription in 4 of 12 human thymus tissue specimens. Gotter *et al.* also reported the existence of Tg expression in medullary epithelial cells of human thymus [25, 26].

The other 4 patients with only mediastinal uptake in our study were not assigned further treatment. The CT scans of thymus and periodic monitoring suggested that they are free of disease, i.e., TSH-suppressed Tg ≤ 2 ng/mL for more than 6 months, though their serum stimulated Tg levels remained increased in the follow-up. It is of note that the degree of angiogenesis in the hyperplastic thymus in TENIS syndrome patients was closely correlated to the elevated serum Tg levels (Pearson *r* = 0.939, *P* = 0.018). This relationship supports our hypothesis that the thymus is a benign origin of the elevated Tg concentrations in TENIS syndrome patients. However, no correlation was found between the volume of the thymus and the Tg concentrations, which is similar to previous FDG studies [27]. Thus, we concluded that it might be the upregulated angiogenesis, not hyperplasia in thymus that affect the secretion of Tg in the enlarged thymus.

Four of the 9 patients with TENIS syndrome had ^99m^Tc-3PRGD2 uptake inside and outside the thymus, and received lymphadenectomy followed by radio-iodine treatment. The serum levels of Tg in these 4 patients were decreased after iodine ablation, but the levels did not drop below the expected threshold. Their stimulated Tg levels remained moderately elevated in the follow-up. Based on previous reports and our data, it could be hypothesized that the activated thymus kept on hypersecreting hormones, including Tg, in the patients during follow-up. The upregulated angiogenesis in thymus provides adequate blood supply to maintain its hypersecretion [28–30]. Actually, a positive correlation was still found between the angiogenesis condition of thymus and the Tg levels in the 9 TENIS syndrome patients when the contribution of the metastatic lesions in 4 patients with lymph node metastasis was tentatively excluded (Pearson *r* = 0.751, *P* = 0.020, *n* = 9).

The incidence of thymus visualization on ^131^I scintigraphy has been estimated to be between 3.4 and 26.3% in different series [5, 31, 32–35]. It has been indicated that the acquired “thyroid follicle-like” structure that exists in the thymus of DTC patients after iodine treatment might be responsible for thymic epithelial cell iodine uptake [5, 32]. As reported in some studies, thymic iodine uptake was much more commonly observed in the subgroup of DTC patients without Tg elevation, and the elevated Tg level in DTC patients who showed only mediastinal uptake on iodine WBS was decreased to normal range after iodine treatment [33, 34, 36]. It is noteworthy that the patients with increased Tg in our study demonstrated a negative radioiodine uptake on ^131^I WBS, even though the increased angiogenesis was determined in their hyperplastic thymus tissue by the ^99m^Tc-3PRGD2 SPECT/CT. Moreover, as we mentioned above, their stimulated Tg levels did not drop back to lower than 2 ng/mL after radio-iodine treatment. Therefore, it could be concluded that the hyperplastic thymus in the patients with TENIS syndrome may not be as differentiated as the thymic tissues seen on the ^131^I WBS, which is considered to possess the property of uptaking iodine [35]. This kind of de-differentiated thymus tissue might keep on producing Tg, or Tg like secretions in the DTC patients [37].

Our results, for the first time, indicated that there is an increased prevalence of angiogenesis in thymic hyperplasia of the patients with DTC. We also found that the degree of angiogenesis in the hyperplastic thymus was positively correlated with the elevated serum Tg level. The Tg levels would not fall back to normal even after an ablative radioiodine therapy dose had been administered. The understanding of the ^99m^Tc-3PRGD2 imaging appearance of thymic hyperplasia and its association with increased serum Tg level is important for preventing misdiagnosis of metastatic disease and avoiding unnecessary treatment, such as surgery or radioiodine therapy [38, 39]. Further longitudinal studies are needed to confirm that the hyperplastic thymic with activated angiogenesis does not necessarily herald recurrence and metastasis of DTC [40].

This study is limited by a selection bias in that only a small number of TENIS syndrome patients were included and were only followed for a limited period. Thus, it may not be possible to generalize our results to all DTC patients. To further clarify this issue, we are beginning a prospective, longitudinal study that will include DTC patients after radioiodine ablation at our institution, thus avoiding the selection bias.

In summary, our study indicated that thymic hyperplasia was commonly observed in thyroid cancer patients, and the hyperplastic thymus with activated vasculature was strongly related with consistently elevated Tg levels in the follow-up of our patients. Based on the existing literature and our data, we propose further intervention for patients with outside thymus uptake of RGD, while close follow-up for patients with only mediastinal uptake.

## MATERIALS AND METHODS

### Subjects

Between January 2015 and December 2016, 763 DTC patients were referred to the Department of Nuclear Medicine at The First Affiliated Hospital of Xi’an Jiaotong University for adjuvant radioiodine treatment after total thyroidectomy. Then they were followed up with serial Tg tests and imaging measurements including neck ultrasound (US), CT scans, and diagnostic dosage ^131^I WBS [1, 2]. Among these patients, 30 cases presented with increased Tg (>2 ng/mL) on thyroid-stimulating hormone (TSH) stimulation and negative results in radiological tests in the follow-up enrolled in for a ^99m^Tc-3PRGD2 SPECT/CT [41].

^131^I WBS was obtained with a large field gamma camera, whole body and high energy collimator, 48 h after the administration of oral diagnostic dose of 185MBq ^131^I to each patient. CT scans were performed using a 64-channel CT scanner [Philips (China) Investment Co., Ltd.], and all scans were performed from the cervix to thorax. Serum TSH, Tg, and anti-Tg antibody levels were measured by radioimmunoassay (GASK-PR, CIS-Bio International, subsidiary of Schering S.A., Gif-sur-Yvette, France).

None of the patients had circulating anti-Tg antibodies detected in this study. All the subjects signed informed consent documents. The protocol for this study was in accordance with the Declaration of Helsinki and its subsequent revisions, and it was approved by the Institutional Review Boards of the First Affiliated Hospital of Xi’an Jiaotong University.

### ^99m^Tc-3PRGD2 SPECT/CT imaging

As mentioned, 30 DTC cases with TENIS syndrome were enrolled in for a ^99m^Tc-3PRGD2 SPECT/CT. Synthesis of the labeling precursor, kit preparation and subsequent ^99m^Tc-labeling were performed as previously described [21]. ^99m^Tc-3PRGD2 SPECT and coregistered CT were performed 0.5 h after intravenous injection of 11.1 MBq/kg (0.3 mCi/kg) of ^99m^Tc-3PRGD2. The Dicom image files of each patient were saved in optic discs and transferred to a Symbia T16 workstation (Siemens, Germany) for analysis.

**Figure 6:**
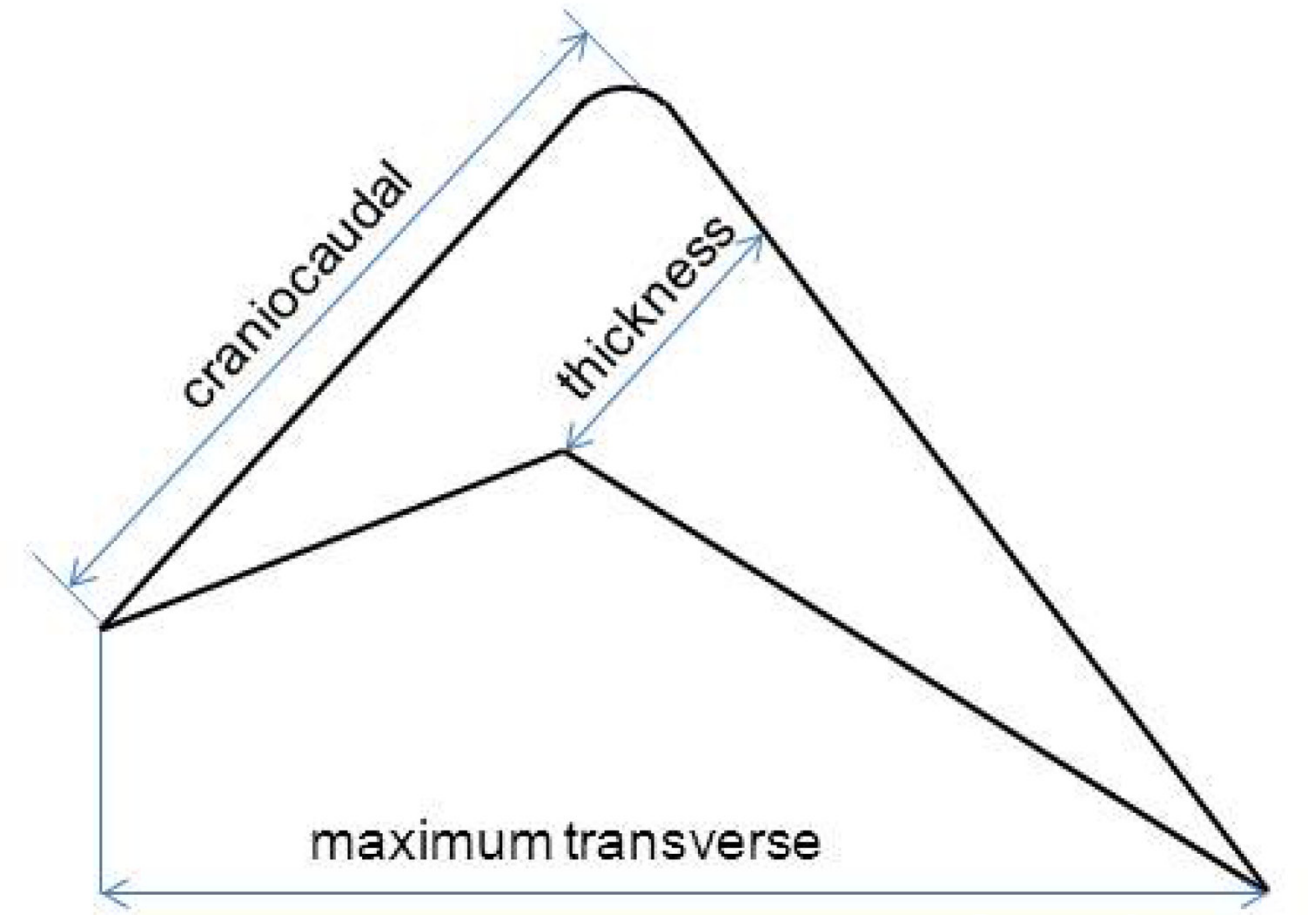
Measurement method of the thickness, craniocaudal and maximum transverse dimensions of the thymus gland

^99m^Tc-3PRGD2 SPECT images were visually interpreted by the consensus of three experienced nuclear medicine physicians with reference to SPECT/CT fusion and CT images. For each case, the mediastinum was reviewed on soft-tissue window settings in the co-registered diagnostic CT (window level = 50, window width = 350). For cases that met criteria for thymic hyperplasia, medical imaging records were reviewed [36]. Measurements of the size (craniocaudal extent, maximum transverse dimensions and thickness, Figure 6), shape (triangular, arrowhead, round, or quadrilateral), margin (smooth or multi-lobular), and internal characteristics of structures (homogeneous or inhomogeneous because of fatty infiltrations) of the thymus were analyzed using an electronic caliper measuring device available on our PACS software [31, 42]. The ^99m^Tc-3PRGD2 uptake in the thymus was also analyzed. For semi-quantitative analysis, tumor-to-background (T/B) ratios of the SPECT/CT images were measured by the same physicians using a standardized method as described below. A volume-of-interest method was used to obtain the maximum values of the enlarged thymus in mediastinal region. The blood pool of the aortic arch was set as control [43].

### Follow-up

The SPECT/CT findings were compared with histopathological findings, serial radiological or clinical follow-up [43]. In patients with only stable thymus enlargement on concomitant imaging, no therapeutic intervention was initiated, whereas patients with other metastatic lesions received surgery and/or an empirical ^131^I treatment. Mediastinal dissection was carried out in patients showing progressive thymus enlargement. Patients were considered free of disease if there were no clinical or biochemical signs of recurrence, i.e., Tg ≤ 2 ng/mL accompanied by negative diagnostic ^131^I WBS under 3–4 weeks TSH stimulation, or Tg ≤ 2 ng/mL for at least 6 months (evaluated every 3 months) under thyroid hormone suppression therapy [5].

### Statistical analysis

Data are shown as the mean ± SD or median (range), as appropriate. To identify individual patients in the current study, we assigned each patient a unique number, which is used throughout the text, tables, and figures. Pearson correlation analysis was performed to assess the linear relation between T/B ratios and the serum Tg levels. A *P*-value of less than 0.05 was considered statistically significant.

## Abbreviations

TENIS: Tg-elevated Negative Iodine Scintigraphy
T/B: Tumor/Background
Tg: Thyroglobulin
DTC: Differentiated thyroid cancer
^131^I WBS: ^131^I Whole-body scan
SPECT/CT: Single photon emission computed tomography/ X-ray computed tomography
TSH: Thyroid-stimulating hormone
T/B: Tumor-to-background
